# Strategies for recruitment and retention of underrepresented populations with chronic obstructive pulmonary disease for a clinical trial

**DOI:** 10.1186/s12874-019-0679-y

**Published:** 2019-02-21

**Authors:** Beatrice Huang, Denise De Vore, Chris Chirinos, Jessica Wolf, Devon Low, Rachel Willard-Grace, Stephanie Tsao, Chris Garvey, Doranne Donesky, George Su, David H. Thom

**Affiliations:** 1 Department of Family and Community Medicine, University of California San Francisco, 995 Potrero Avenue, Building 80, Ward 83, San Francisco, CA 94110 USA; 2 Department of Family and Community Medicine, University of California San Francisco, San Francisco, CA USA; 3 Department of Family and Community Medicine, University of California San Francisco, San Francisco, CA USA; 4 Department of Family and Community Medicine, University of California San Francisco, San Francisco, CA USA; 5 Department of Family and Community Medicine, University of California San Francisco, San Francisco, CA USA; 6 Department of Family and Community Medicine, University of California San Francisco, San Francisco, CA USA; 7 San Francisco Department of Public Health, San Francisco, CA USA; 8 University of California San Francisco at Mount Zion Sleep Disorders Center, San Francisco, CA USA; 9 Department of Physiological Nursing, University of California San Francisco, San Francisco, CA USA; 10 Department of Medicine: Pulmonology, Critical Care, Allergy and Sleep Medicine Program, University of California San Francisco, San Francisco, CA USA; 11 Department of Medicine, Division of Primary Care and Population Health, Stanford University, Palo Alto, CA USA

**Keywords:** Vulnerable populations, Research design, Patient selection, Chronic obstructive pulmonary disease

## Abstract

**Background:**

Recruitment and retention are two significant barriers in research, particularly for historically underrepresented groups, including racial and ethnic minorities, patients who are low-income, or people with substance use or mental health issues. Chronic obstructive pulmonary disease (COPD) is the third leading cause of death and disproportionately affects many underrepresented groups. The lack of representation of these groups in research limits the generalizability and applicability of clinical research and results. In this paper we describe our experience and rates of recruitment and retention of underrepresented groups for the Aides in Respiration (AIR) COPD Health Coaching Study.

**Methods:**

A priori design strategies included minimizing exclusion criteria, including patients in the study process, establishing partnerships with the community clinics, and ensuring that the health coaching intervention was flexible enough to accommodate patient needs.

**Results:**

Challenges to recruitment included lack of spirometric data in patient records, space constraints at the clinic sites, barriers to patient access to clinic sites, lack of current patient contact information and poor patient health. Of 282 patients identified as eligible, 192 (68%) were enrolled in the study and 158 (82%) completed the study. Race, gender, educational attainment, severity of disease, health literacy, and clinic site were not associated with recruitment or retention. However, older patients were less likely to enroll in the study and patients who used home oxygen or had more than one hospitalization during the study period were less likely to complete the study. Three key strategies to maximize recruitment and retention were identified during the study: incorporating the patient perspective, partnering with the community clinics, and building patient rapport.

**Conclusions:**

While the AIR study included design features to maximize the recruitment and retention of patients from underrepresented groups, additional challenges were encountered and responded to during the study. We also identified three key strategies recommended for future studies of COPD and similar conditions. Incorporating the approaches described into future studies may increase participation rates from underrepresented groups, providing results that can be more accurately applied to patients who carry a disparate burden of disease.

**Trial registration:**

This trial was registered at ClinicalTrial.gov at identifier NCT02234284 on August 12, 2014.

Descriptor number: 2.9 Racial, ethnic, or social disparities in lung disease and treatment.

## Background

Despite a concerted effort by the National Institutes of Health [1], other federal agencies [2], and individual investigators [3], problems persist in the recruitment and retention of patients from underrepresented populations, including racial and ethnic minorities, non-English speakers, and patients who are low-income, have low health literacy, or have substance use or mental health problems [4–7]. Lack of representation from these groups limits the generalizability of research findings [8–10] and may contribute to ongoing disparities in the provision of health care and inequities in health policy and funding decisions. Underrepresentation also limits the ability to tailor treatment to the populations who often carry the highest burden of disease [11]. To address such disparities it is important to develop well-designed, culturally adapted research interventions and health promotion programs that are relevant to the population of interest [12].

Challenges to recruiting and retaining members of underrepresented groups include choosing research questions relevant to these groups and establishing relationships with communities being recruited [13–15]. In addition, members of underrepresented groups may lack experience with or knowledge about medical research [11] and face practical barriers to participation such a longer travel times and less flexible schedules [15–17]. They also may have beliefs and practices that may contrast with some elements of the study design [12, 18]. Minority groups are also more likely to have co-morbidities, which may result in their exclusion from, and lower participation in, clinical trials [19].

Disparities in the prevalence and severity of COPD and in access to care for low-income and racial or ethnic minority patients is well documented [20–26]. Improving the inclusion of patients from these ‘at risk’ populations in COPD research is needed to address these disparities. While there is limited research on recruitment and retention of underrepresented groups for studies of people with asthma [15, 27] there is little, if any, such research for people with COPD.

In designing and conducting a randomized controlled trial of health coaching for patients with moderate to severe COPD cared for in urban public health “safety-net” primary care clinics, we faced challenges to recruiting and retaining patients who were members of racial and ethnic minority groups, had low English proficiency, and/or had concurrent co-morbid, substance use or mental health conditions. In this paper, we report study design features intended to enhance recruitment and retention, as well as challenges to recruitment and retention that arose during the study, strategies used to meet these challenges, and rates of recruitment and retention of study.

## Methods

### The Aides in Respiration (AIR) study

The AIR study has been previously described [28]. In brief, the AIR study is a randomized controlled trial comparing 9 months of health coaching to usual care for urban, low-income patients with moderate to severe COPD. The AIR study was funded by Patient Centered Outcomes Research Institute (PCORI) and the study protocol was approved by the UCSF Human Research Protection Program (Approval #14–12,872) and registered with ClinicalTrials.gov (NCT02234284).

### Design features to increase recruitment and retention from underrepresented groups

#### Setting and patient population

Between November 12, 2014 and May 6, 2017, the AIR study enrolled patients from seven urban public health based primary care clinics that serve a diverse group of low-income patients that are often underrepresented in clinical trials. Patients were eligible if they were age 40 and older, spoke English or Spanish, and had moderate to severe COPD. We did not require patients to identify as having COPD, as long as they recognized they had an ongoing lung condition or breathing problem. To maximize participation of underrepresented groups, we minimized exclusion criteria. Specifically, we did not exclude patients who were homeless, had active substance use, mental illness, or in poor health as long as they were able to receive health coaching and participate in the study.

#### Community clinic partners

In designing the study, we worked with community-based clinics, initially contacting and meeting with clinic leadership (medical director and staff and nursing managers) to identify benefits of the study to the clinic, address concerns, and establish protocols to minimize impact on clinic function. Benefits to the clinic included providing spirometry and exercise capacity results to clinicians for all patients enrolled in the study. Patients randomized to the health-coaching arm also received education including instructions regarding inhaler use and other support. Clinicians received expert recommendations for COPD management for patients enrolled in the coaching arm. The latter benefit was extended to include patients in the usual care arm at the end of the study. We also provided in-service education to clinical staff and a payment of $1000 to each clinic cover staff time and support. Prior to the beginning of patient enrollment at each site, we attended staff and provider meetings to explain the study, answer questions and address concerns. We also committed to reporting results from the study back to the clinics.

#### Patient involvement

We included patients with COPD from the target study population in planning and execution of the studies. Patient advisors were initially identified through an existing community-based spirometry program. An Advisory Board that included 4 to 6 patients met prior to the beginning and over the course of the study. The study team consulted with the Advisory Board regarding recruitment strategies, survey items, and the content of intervention and retention of participants. We also worked with a patient partner who participated in the health coach training and provided advice to health coaches throughout the study.

#### Patient incentives

Benefits to patients included additional information about their disease by spirometry and 6-min walk test at enrollment and 9 months, and expert review of their treatment plan either during the study (for those randomized to health coaching) or after the study (for those in the usual care group). Patients received monetary compensation of up to $30 for completing survey and testing at enrollment, $10 for answering surveys at 3 and 6 months and $60 for the end of study survey and testing. In addition, we committed to providing a forum for reporting results from the study back to interested patients.

#### Research staff training

Both health coaches and one research assistant were bilingual in Spanish and English. Both research assistants had at least five years’ experience of working in a safety net setting. Research assistants and coaches received training that included education around the physiology of COPD, comprehensive review of inhalers with particular emphasis on observing technique, Global Initiative for Chronic Obstructive Lung Disease (GOLD) guidelines, and management of COPD. Research assistants received in-depth training on how to conduct spirometry with the Director of the Community Spirometry program and were required to demonstrate competency before attempting spirometry with patients. Observations of research assistants in administering the survey, spirometry, and 6-min walk tests were conducted at multiple time points for quality assurance.

#### Study materials

Study materials, including informed consent, testing instructions and survey questions were available in both English and Spanish and were written at a 5th grade reading level. To maximize comprehension for patients with low literacy, research assistants typically read written materials unless the patient indicated they preferred to read for themselves. Study survey questions were piloted prior to the study and modified if needed for clarity or to reduce respondent burden.

#### Health coaching intervention

The health coaching intervention was individualized, supportive and flexible. The intervention for our study required the patient to be willing to work with a health coach. While the health coaching was structured in terms of activities, goals and techniques, the delivery was quite flexible and allowed contacts by phone or at a place and time most convenient to the patient. Timing and location of in-person visits depended on what was convenient for the patient. Visit locations included, but were not limited to, clinic (as a stand-alone visit or in conjunction with a medical visit), the patient’s home, libraries, coffee shops, or some other site within the patient’s neighborhood.

#### Continuity with the same member of the study team

Emphasis was placed on having a continuous relationship with the study team by having the same research assistant conduct all baseline and follow up measures and act as the point of contact for the patient. Also, the study design was intended to ensure that contact would be maintained with the patient at least every three months. Research assistants were also provided a cell phone by which patients could contact them directly.

A list of design features intended to enhance recruitment and retention of underrepresented groups is summarized in Table 1 [29–46].

**Table 1:** Study design characteristics to support enrollment and retention of underrepresented minorities

Area of focus	Design Characteristic
Community interaction	Conducting study in safety net community clinics [29–31]
	Working with community-based patient partners [13, 14, 18, 29, 32]
	Partnering with community clinics [29, 30, 32]
	Meeting with patients and stakeholders over course of study (study advisory board) [33–38]
Clinic interaction	Creating value for clinical care (e.g., providing test results to primary care clinicians) [13, 30, 33]
	Providing education to clinic staff [33]
	Payments to clinics [40]
	Basing study activities at community clinics [37, 38, 40–43]
Patient interaction	Providing information to patient about their condition [13]
	Bilingual staff and study materials [14, 18, 34]
	Training research staff in outreach, recruitment and retention, especially for minority and underserved populations [45]
Recruitment	Face to face recruitment and enrollment when possible [8, 14]
	Minimal inclusion/exclusion criteria [19]
	Not requiring patient to endorse diagnosis of condition [14, 27]
Retention	Meeting with patient at home or close to where patient lived [14, 18]
	Continuity of personal relationship with member of study team [8, 18, 46]
	Maintaining contact at 3 month intervals [14, 44]
Both recruitment and retention	Flexibility in rescheduling meeting times [14, 18]
	Providing compensation to patients [8, 13, 14, 27, 44]

## Results

### Challenges to recruitment and retention encountered during the study

Though significant efforts of planning and preparations were done to optimize recruitment, once recruitment officially started, unforeseen challenges came up. These challenges are described below and in Table 2 and discussed below.

**Table 2:** Challenges faced during the study and solutions

Challenges	Solution
Lack of spirometric data by which to identify eligible patients	Worked in conjunction with the spirometry community program Trained research assistants to perform post-bronchodilator spirometry Looked for other markers of COPD to help with identification of potentially eligible patients (ex. medications, exacerbation history) Multiple modes of recruitment including in-person during a scheduled visit, phone calls, letters, flyers in clinic
Limited patient access due to poor health and transportation barriers	Flexibility in scheduling, accepting no shows and rescheduling, meeting patients closer to home, meeting with patients during their medical visits, home visits
Space constraints at clinic	Scheduling around times where space is available, using more than one visit if space not available for sufficient time, meeting patients outside of the clinic setting
Diverted attention	Prioritization of recruitment
Loss to follow up (by phone)	Other forms of communications used (letters, emergency contacts)
Poor patient health	Home visits

#### Lack of spirometric data in the patient record

A formal diagnosis of COPD requires clinical suspicion and post-bronchodilator spirometry showing obstruction (FEV1/FVC < .70). Spirometry also provides a measure of COPD severity. Of 1881 patients in our target population who had one or more visits coded as COPD, emphysema, or chronic bronchitis, 78% (*n* = 1218) did not have any spirometry in their record at all. The lack of spirometry made the identification of eligible patients more challenging and required the study team to often conduct post-bronchodilator spirometry to establish a diagnosis of COPD. Working in conjunction with the community spirometry program, research assistants were trained to conduct post-bronchodilator spirometry. Rather than having to schedule the patients in the pulmonary function testing lab with long wait times, spirometry was brought to the patients’ home clinics.

#### Barriers to patients’ access to the study

Community clinics aim to serve their immediate neighborhood, but patients often live at greater distances. Of the enrolled patients with a reported zip code (*n* = 188), 70% (*n* = 132) lived outside of their primary care clinic zip code. The burden of travel was greater for patients with COPD symptoms. Flexibility in scheduling, accepting no shows and rescheduling, meeting patients closer to their home, coordinating meeting patients with their medical visits, and home visits were all done to address this challenge.

#### Scheduling and space constraints

Dedicated space for spirometry and the 6-min walk test was available to the study team at only two out of the seven sites. For other sites, limited space resulted in restricted recruitment hours, which did not necessarily align with patients’ availability. In addition, a short recruitment timeframe at each site did not allow accommodation to the patients’ ability to participate if they were experiencing a major life event at time of recruitment. In working with the limited space and time constraints allotted by clinics, the study team had to be flexible with scheduling for the patients. This included breaking the longer visit into two or three shorter ones and meeting patients outside of the clinic when space was not available in the clinic.

#### Competition between recruitment and retention

The unforeseen recruitment challenges pushed the study timeline back, which meant that there was significant overlap between recruitment and 9-month follow up. As such, efforts originally intended to be directed towards retention were instead heavily focused on recruitment. To reach recruitment goals, recruitment visits sometimes took priority over 9-month follow up visits.

#### Lack of current contact information

While the ability to be contacted by phone was required for study enrollment, phones often went in and out of service and numbers were changed. Over the course of the study, 50 patients could not be reached due to having non-working or wrong numbers. To compensate for difficulties in contacting patients via phone, alternate forms of communications, such as letters, use of emergency contacts, or meeting patients at appointments, were utilized.

#### Poor patient health

Some patients’ health declined over the course of the study, making it more difficult to meet at their clinic. The number of home visits conducted increased in frequency to accommodate these needs. Even amongst those who were able to meet to complete the follow up, spirometry and 6-min walk tests were often not performed due to physical limitations of the patients. There were 19 patients who completed spirometry at baseline and 21 patients who completed the 6-min walk test at baseline were not able to do so at 9 months due to health-related reasons.

#### Additional resources required

Recruitment and retention of underrepresented minorities required more staff time and resources to make additional phone calls, reschedule appointments, and travel to meet with patients who have difficulty traveling. [18, 31] than we anticipated. As a result, we needed to extend our recruitment period and re-budget personnel time to meet our goals.

### Rates of recruitment and retention

Of the 282 patients identified as eligible for the study, 192 (68%) were recruited, meeting our revised recruitment goal of 190. In general, patients enrolled were similar to patients known to be eligible but not enrolled, other than being older on average (66 vs 62 years) (Table 3). Of the 192 patients enrolled, 171 (89.1%) belonged to one or more of the underrepresented groups and 62 patients (32.5%) belonged to more than two of these groups. Specifically, over half (57%) were African American and 17% Latino. Over a quarter (29%) reported substance use, 16% had significant symptoms of depression, and approximately a third (32%) had less than a higher school education with a slightly larger proportion (37%) reporting limited health literacy (Table 4). Spirometry was obtained at baseline for 154 enrolled participants (80%), while 134 (70%) were able to complete the 6-min walk test.

**Table 3:** Characteristics, from review of health record, of patients enrolled compared to all eligible patients

	Enrolled (*n* = 192)	Eligible, not enrolled (*n* = 90)	*P*-value
Age, in years, mean (sd)	61.6 (7.6)	65.9 (9.1)	*p* < .01
Male, % (n/N)	68% (125/192)	71% (63/90)	*p* = .42
Primary language other than English, % (n/N)	10.7% (18/169)	8.1% (7/86)	*p* = .52
> 1 hospitalization in 12 months prior to recruitment, % (n/N)	30.2% (58/192)	31.1% (28/90)	*p* = .88
Uses oxygen at home, % (n/N)	6.3% (12/190)	12.2% (11/90)	*p* = .09
Prescription for LAMA or LABA+ICS*, % (n/N)	78.7% (151/192)	84.4% (76/90)	*p* = .25

* *LAMA* Long-acting muscarinic agent; *LABA* Long-acting beta-antagonist; *ICS* Inhaled corticosteroid

**Table 4:** Characteristics of patients retained compared to all patients enrolled

	Enrolled (n = 192)	Completed study (*n* = 158)	Did not complete study (*n* = 34)	P-value*
At enrollment				
Age, in years, mean (sd)	61.3 (7.6)	61.3 (7.5)	61.1 (8.5)	*p* = .92
Male, % (n/N)	65.5 (126/192)	63.9% (101/158)	73.5% (25/34)	*p* = .29
Preferred Language				
English, % (n/N)	87.4% (167/191)	86.6% (136/157)	91.2% (31/34)	*p* = .47
Spanish, % (n/N)	9.4% (18/191)	10.2% (16/157)	5.9% (2/34)	*p* = .44
Other, % (n/N)	3.1% (6/191)	3.2% (5/157)	2.9% (1/34)	*p* = .94
Race				
African-American, % (n/N)	56.7% (109/192)	55.7% (88/158)	61.8% (21/34)	p = .52
White, % (n/N)	21.3% (41/192)	20.9% (33/158)	23.5% (8/34)	*p* = .73
Asian, % (n/N)	3.6% (7/192)	4.4% (7/158)	0.0% (0/34)	*p* = .21
Other, % (n/N)	16.1% (31/192)	19.0% (30/158)	14.7% (5/34)	*p* = .56
Ethnicity- Latino/Hispanic, % (n/N)	16.7% (32/192)	17.7% (28/158)	11.8% (4/34)	*p* = .40
Substance use, % (n/N)	28.6% (55/192)	26.6% (42/158)	38.2% (13/34)	*p* = .17
PHQ score ≥ 15, % (n/N)	15.7% (30/192)	16.6% (26/158)	11.8% (4/34)	*p* = .49
< High school education, % (n/N)	31.9% (61/191)	31.9% (50/157)	32.4% (11/34)	*p* = .95
Limited health literacy**, % (n/N)	37.2% (71/191)	39.5% (62/157)	26.5% (9/34)	*p* = .15
FEV1 < 50% predicted	38.8% (59/152)	38.0% (49/129)	43.5% (10/23)	*p* = .62
Gold category D, % (n/N)	46.3% (88/190)	45.2% (71/157)	51.5% (17/33)	*p* = .51
During study period				
> 1 hospitalization, % (n/N)	25.0% (48/192)	22.2% (35/158)	38.2% (13/34)	*p* < 0.05
> 1 hospitalization for COPD, % (n/N)	18.2% (35/192)	17.7% (28/158)	20.6% (7/34)	*p* = .69
Uses oxygen at home, % (n/N)	18.3% (35/191)	14.7% (23/157)	35.3% (12/34)	*p* < 0.005

*For completed study vs did not complete study

**Needs someone to help read medial information at least a little of the time

The study retained 158 patients (82%) which met our goal of at least 80%. As shown in Table 4, the only patient characteristics that appeared to predict loss to follow-up were use of home oxygen at baseline and having more than one hospitalization during the study period. Patients using oxygen likely had greater clinical symptoms and disease severity, which would affect their ability to travel to clinic and complete the measures required for completion of the study. There was no significant association between clinic site and likelihood of being lost to follow-up.

## Discussion

The AIR study employed a variety of techniques around stakeholder engagement, community partnering, building trust with patients and community and creating value for patients and clinics to maximize enrollment and retention of patients often underrepresented in clinical research due to economic, language and cultural barriers, or co-existing mental health, substance use or co-morbid disease. Our rates of recruitment (68%) and retention (82%) compare favorably to previous randomized controlled trials of similar interventions, including those with more extensive exclusion criteria. In three community-based studies which recruited patients hospitalized for COPD exacerbation, recruitment rates were 22%, 47%, and 50% and retention rates were 65%, 89%, and 92%, respectively [47–49]. Another RCT of self-management support that recruited patients from primary care and specialty clinics in a large academic center reported a recruitment rate of 41% and a retention rate of 81% [50–52]. Two studies that recruited from patients referred for pulmonary rehabilitation or to a COPD nurse, reported recruitment rates of 54% and 84%, respectively, and retention rates of 51% and 81% [53, 54]. Another study, which recruited patients at the time they filled a prescription for a COPD medication, reported enrolling 69% of eligible patients with 95% completing the study [55]. Notably, several studies had exclusion criteria that would have disqualified many patients from the AIR Health Coaching Study; for example, having diagnosis of other lung diseases, unstable cardiac, renal, or hepatic disease [49] or having mental disorders, including schizophrenia, dementia, alcohol or drug abuse [54].

While it is not possible to precisely link which features of the AIR study were most important for recruitment and retention, we believe, based on our experience in the AIR Study, that three strategies- incorporating patients’ perspectives, establishing community partnerships, and building rapport with patients - were key to reaching our goals for recruitment and retention. Each strategy is described below.

### Incorporating patients’ perspectives

This study made a concerted effort to incorporate patients’ perspectives in multi-faceted approaches. As mentioned previously, the establishment of the advisory board, early discussions with patients from the community spirometry program, and most importantly, having a patient partner as a study team member throughout the entirety of the study helped the team view everything from the patient perspective. Having this perspective gave a realistic view of what was feasible or appropriate for the study population, such as survey length or how and where to interact with patients. Changes based on recommendations from patients made this study more sensitive to the needs and perspectives of the study population and may have helped the study avoid some of the stigma often associated with research among underrepresented groups.

### Partnering with community sites

Our study experience corroborates reports by others indicating that partnering with community clinics is vital to enrollment and retention of underrepresented populations [56]. The community clinics with which we partnered are highly mission driven, so communicating the potential benefits of the study to patients (e.g., obtaining important clinical tests such as spirometry or the 6-min walk test, facilitating specialist review of cases, and providing health coaching to half of those enrolled) was critical in messaging. The study team also demonstrated their commitment to the partnership by supporting initiatives of the community clinics, such as participating in health fairs or offering in-service sessions and attending clinic huddles or holiday events. At the beginning of the study, the team committed to visiting each interested site to report on findings or offer additional training to support COPD patients after the end of data collection. The depth of this reciprocal commitment was vital to overcoming barriers of resource limitations that could otherwise have hobbled the study (e.g., finding space on busy days).

### Building rapport with patients

Rapport is defined as a close and harmonious relationship in which the people or groups concerned are ‘in sync’ and is considered a therapeutic relationship between a doctor and patient [57]. The initial appointments at baseline, lasting 2–3 h, allowed time for the research staff to build trust and rapport with participating patients. This time gave patients space to talk and ask questions. It also allowed the research staff to take their time in explaining the study, going through the survey and other measures, and allowed for breaks if needed. Having the same staff member from baseline reach out to the patient for follow up attempts may have promoted the likelihood of them showing up for follow up, which ultimately contributed to the retention rate. Establishing rapport with patients also helped to decrease the stigma often associated with research. Once patients felt they could trust the research staff, they were highly motivated to participate in the study and actually encouraged other patients to participate. Thus building rapport with individuals led to building rapport with the community. At the end of the study, all study participants and stakeholders were invited to a gathering to celebrate the successful completion of the study and to report results.

These three strategies are feasible, though each requires early planning and an investment of resources to implement. Incorporating patients’ perspectives requires identifying and meeting with patients, caregivers, and patient advocates. Often potential patient representatives can be identified by members of the clinic staff or from groups such as clinic advisory boards or disease-specific education and support groups. It is also important to advertise more widely to give patients a chance to self-nominate. Covering the cost of participation (e.g., transportation, childcare) and at least a nominal incentive is also important. The second strategy, developing a relationship with community partners such as clinic sites, also requires a sustained longitudinal commitment, preferably starting prior to the actual study. In our experience, most community clinic sites are committed to their patients and their patients’ communities. Participation in a research study may take resources, distract from patient care, or be perceived negatively by the community. Developing a positive relationship with one or more member of the clinic administrative and service staff early on is critical. Offering to help with existing activities such as health fairs or community outreach and providing educational or other resources can help build a relationship. Engaging clinic leadership in identifying ways the clinic could benefit from the study, and how to minimize the adverse impact of the study, is also important. The third strategy, building rapport with patients, can be rewarding for study staff and patients alike and does not require many additional resources. Choosing staff who have experience working with the study’s target groups is helpful, though not absolutely necessary. Providing opportunities for frequent contact between patients and the same member of the staff can help facilitate rapport.

### Limitations

The lack of data available for those not enrolled in this study limits our ability to characterize these people are, what barriers they may face, and to identify strategies targeted to increase the representation of this population into research. This study was not designed to test different recruitment strategies. Thus, our results are primary descriptive and our judgements about the relative importance of patient participation, community partnerships, and building rapport are based on our experience.

## Conclusions

While the AIR study included design features to maximize the recruitment and retention of patients from underrepresented groups, additional challenges were encountered and responded to during the study. Some challenges were specific to patients with pulmonary disease, such as the lack of spirometric testing. Others, such as poor health which limited access to the study, can be expected in other moderate to severe conditions. Still others, including lack of space at the clinic sites at which to meet with patients and limited patient contact information, can be found in many clinics serving underrepresented groups of patients. We hope that reporting our experience and the three key strategies we describe will help improve future studies of COPD and similar conditions. Adopting these approaches could help investigators achieve participation rates of at least 70%, providing results that can be more accurately applied to patients from underrepresented groups, who carry a disparate burden of COPD.

## Abbreviations

AIR: Aides in Respiration
COPD: Chronic obstructive pulmonary disease
FEV: Forced expiratory volume
FEV1: Forced expiratory volume at 1 s
GOLD: Global Initiative for Chronic Obstructive Lung Disease
ICS: Inhaled corticosteroid
LABA: Long-acting beta-antagonist
LAMA: Long-acting muscarinic agent
PCORI: Patient Centered Outcome Research Institute
